# Targeting the prefrontal-supplementary motor network in obsessive-compulsive disorder with intensified electrical stimulation in two dosages: a randomized, controlled trial

**DOI:** 10.1038/s41398-024-02736-y

**Published:** 2024-02-05

**Authors:** Jaber Alizadehgoradel, Behnam Molaei, Khandan Barzegar Jalali, Asghar Pouresmali, Kiomars Sharifi, Amir-Homayun Hallajian, Vahid Nejati, Benedikt Glinski, Carmelo M. Vicario, Michael A. Nitsche, Mohammad Ali Salehinejad

**Affiliations:** 1 Department of Psychology, Faculty of Humanities, University of Zanjan, Zanjan, Iran; 2 Department of Psychiatry and Psychology, School of Medicine, Ardabil University of Medical Sciences, Ardabil, Iran; 3 Department of Psychiatry, School of Medicine, Hacettepe University, Ankara, Turkey; 4 Department of Family Health, Social Determinants of Health Research Center, Ardabil University of Medical Sciences, Ardabil, Iran; 5 Sharif Brain Center, Department of Electrical Engineering, Sharif University of Technology, Tehran, Iran; 6 School of Cognitive Sciences, Institute for Research in Fundamental Sciences, Tehran, Iran; 7 Department of Psychology and Educational Sciences, University of Tehran, Tehran, Iran; 8 Department of Psychology, Shahid Beheshti University, Tehran, Iran; 9 Department of Psychology and Neurosciences, Leibniz Research Centre for Working Environment and Human Factors, Dortmund, Germany; 10 Dipartimento di Scienze Cognitive, Psicologiche, Pedagogiche e degli studi culturali, Università di Messina, Messina, Italy; 11 Bielefeld University, University Hospital OWL, Protestant Hospital of Bethel Foundation, University Clinic of Psychiatry and Psychotherapy and University Clinic of Child and Adolescent Psychiatry and Psychotherapy, Bielefeld, Germany; 12 German Centre for Mental Health (DZPG), Bochum, Germany

**Keywords:** Psychiatric disorders, Neuroscience

## Abstract

Obsessive-compulsive disorder (OCD) is associated with a high disease burden, and treatment options are limited. We used intensified electrical stimulation in two dosages to target a main circuitry associated with the pathophysiology of OCD, left dorsolateral prefrontal cortex (l-DLPFC), and pre-supplementary motor area (pre-SMA) and assessed clinical outcomes, neuropsychological performance, and brain physiology. In a double-blind, randomized controlled trial, thirty-nine patients with OCD were randomly assigned to three groups of sham, 2-mA, or 1-mA transcranial direct current stimulation (tDCS) targeting the l-DLPFC (F3) and pre-SMA (FC2) with anodal and cathodal stimulation respectively. The treatment included 10 sessions of 20-minute stimulation delivered twice per day with 20-min between-session intervals. Outcome measures were reduction in OCD symptoms, anxiety, and depressive states, performance on a neuropsychological test battery (response inhibition, working memory, attention), oscillatory brain activities, and functional connectivity. All outcome measures except EEG were examined at pre-intervention, post-intervention, and 1-month follow-up times. The 2-mA protocol significantly reduced OCD symptoms, anxiety, and depression states and improved quality of life after the intervention up to 1-month follow-up compared to the sham group, while the 1-mA protocol reduced OCD symptoms only in the follow-up and depressive state immediately after and 1-month following the intervention. Both protocols partially improved response inhibition, and the 2-mA protocol reduced attention bias to OCD-related stimuli and improved reaction time in working memory performance. Both protocols increased alpha oscillatory power, and the 2-mA protocol decreased delta power as well. Both protocols increased connectivity in higher frequency bands at frontal-central areas compared to the sham. Modulation of the prefrontal-supplementary motor network with intensified tDCS ameliorates OCD clinical symptoms and results in beneficial cognitive effects. The 2-mA intensified stimulation resulted in larger symptom reduction and improved more converging outcome variables related to therapeutic efficacy. These results support applying the intensified prefrontal-SMA tDCS in larger trials.

## Introduction

With a lifetime prevalence of 2–3%, Obsessive-compulsive disorder (OCD) is one of the most disabling psychiatric disorders [1], with substantial functional impairment and increased risk of early mortality [2, 3]. Individuals with OCD have unwanted and distressing thoughts (obsessions) and repetitive behaviors that the individual feels driven to perform (compulsions) [4]. While cognitive-behavioral therapy with exposure/response prevention and serotonin reuptake inhibitor medication are considered first-line treatments for OCD, up to 40% of patients fail to respond to these treatments [5].

Non-invasive brain stimulation techniques provide unique opportunities to not only study brain functions but also to modify core physiological parameters of human behavior and cognition (e.g., neuroplasticity) in both healthy and clinical populations [6, 7]. Some non-invasive brain stimulation techniques, such as repetitive transcranial magnetic stimulation (rTMS), are Food and Drug Administration (FDA)-approved for the treatment of several major neuropsychiatric disorders, including OCD [8], suggesting that other forms of techniques may be considered as a potential intervention for patients with OCD. Transcranial direct current stimulation (tDCS) is a non-invasive brain stimulation technique that uses a weak direct electrical current to modulate brain activity and excitability [9]. The exact mechanisms by which tDCS works are not fully understood, but its primary mechanism of action, which emerges immediately during stimulation, involves subthreshold de- or hyperpolarization of neuronal membrane potentials, resulting in excitability-enhancing effects by anodal and excitability-reducing effects by cathodal stimulation in conventional protocols [10, 11]. In neuropsychiatric disorders that are characterized by functional brain abnormalities (i.e., hyper- or hypoactivity of specific brain region/s and network/s), it is possible to modify altered brain functions with tDCS and affect target behavior or cognition [12–16]. In OCD, results of tDCS studies have been mixed so far, and knowledge is still limited about optimal stimulation parameters and efficacy of interventions, such as in other clinical non-invasive brain stimulation scenarios [12, 17–20].

Functional abnormalities of the dorsolateral prefrontal cortex (DLPFC) are documented in OCD [21]. Specifically, response inhibition, a core cognitive ability that is severely impaired in OCD is linked to several regions of the prefrontal cortex, including the DLPFC and inferior frontal gyrus [22–24]. Another cortical region that is consistently shown to be involved in the pathophysiology of OCD is the pre-supplementary Motor Area (pre-SMA), which is important for inhibitory control, especially of ongoing actions [25–27]. In OCD patients, the pre-SMA is hyperactive, especially during cognitive task performance that requires attentional and inhibitory control [28, 29], and is, therefore, a major target of non-invasive brain stimulation treatment [17, 30, 31]. Although the left DLPFC and pre-SMA have been targeted in previous tDCS studies, targeting both regions with anodal and cathodal stimulation respectively has not been reported so far [19, 32]. Applying a protocol that can modulate the prefrontal-SMA network and presumably restore physiological abnormalities can have therapeutic effects.

Beyond the choice of the target region, stimulation parameters (e.g., stimulation intensity and repetition) are critical for the efficacy of the neurostimulation intervention, and recent work stress on optimizing and/or individualizing the intervention [33, 34]. Physiological findings in healthy humans have shown that repeated stimulation with a short interval (e.g., two consecutive stimulation sessions with a 20 min interval) can induce long-lasting LTP-like plasticity in the brain [35]. This has implications for the clinical application of tDCS. We recently showed that such a stimulation protocol, which we refer to as “intensified” protocol, has stronger and longer therapeutic effects on social anxiety disorder [36]. In the present study, we adopted the same stimulation protocol and furthermore, included different outcome variables to evaluate treatment efficacy. In addition to primary clinical symptoms, we assessed core cognitive deficits in OCD patients (e.g., response inhibition, working memory) [37] and monitored changes in the oscillatory power spectrum and functional connectivity of the brain, which are abnormally changed in OCD, such as reduced and raised alpha and delta power respectively and reduced functional connectivity [38–41].

Accordingly, in this registered, randomized, double-blind, sham-controlled clinical trial we aimed to (1) investigate the effect of intensified stimulation over prefrontal and pre-SMA regions on primary and secondary clinical variables in patients with OCD, (2) explore the stimulation dosage-dependency (1-mA vs 2-mA) of treatment efficacy, (3) and examine the effects of these interventions on cognitive (response inhibition, attention bias) and electrophysiological (oscillatory power, functional connectivity) correlates of the psychopathology of OCD. This is the first tDCS RCT in OCD to explore the effects of a novel intensified tDCS intervention at two different stimulation intensities on symptom reduction and neurocognitive correlates of OCD.

## Methods

### Participants

**Fig. 1:**
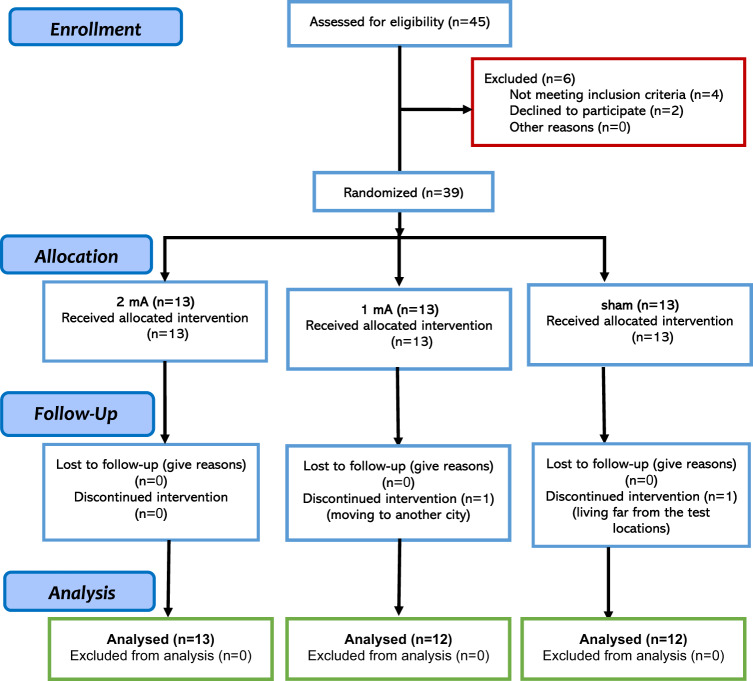
CONSORT flow diagram of study inclusion.

This study had a randomized, double-blind, parallel-group design to prevent blinding failure and carry-over effects. Thirty-nine individuals diagnosed with OCD (mean age = 31.59, SD = 8.24, 26 females) were recruited from several neuropsychiatric clinics in Ardabil, Iran from August 2020 to January 2022. Patients were randomly assigned to the active and sham stimulation groups by the block randomization method (supplementary content). The sample size was calculated a priori based on a medium effect size suggested for tDCS studies [42] (*f* = 0.30, *α* = 0.05, power = 0.95, *N* = 39, mixed-model ANOVA with 3 measurements). Two patients from the 1-mA and sham groups did not complete the whole treatment, and final analysis was conducted on 37 participants (1 mA tDCS *N* = 12, 2 mA tDCS *N* = 13, sham tDCS *N* = 12) (Fig. 1). The inclusion criteria were: (1) diagnosis of OCD according to DSM-5, (2) being 18–50 years old, (3) being non-smoker, (4) no previous history of neurological diseases, brain surgery, epilepsy, seizures, brain damage, head injury, or metal brain implants, and (5) absence of other psychiatric disorders. Those patients taking anxiolytic (*N* = *6*) and/or SSRI (*N* = 22) medication were receiving stable doses for 6 weeks before the experiment up to the follow-up. All participants were native speakers and had normal or corrected-to-normal vision. This was a registered clinical trial (ClinicalTrials.gov Identifier: NCT05501132) approved by the Ethics Committee of the Ardabil University of Medical Science (Ethics code: IR.ARUMS.REC.1399.102). Participants gave their written informed consent before participation (see Table 1 for demographics).

**Table 1:** Demographic data.

	1-mA tDCS	2-mA tDCS	sham tDCS	*p* value*
Sample size (*n*)	12	13	12	
Age – Mean (SD)	33.91 (9.52)	28.46 (7.04)	32.66 (7.67)	0.224
Sex – Male (female)	4 (8)	3 (10)	4 (8)	0.809
Marital status				
Single	6	6	6	0.221
Married	6	8	7	
Divorced	0	1	1	
Time since diagnosis (years)- mean (SD)	4 (1.85)	3.50 (1.78)	4.16 (2.24)	0.851
On medication (*n*)	9	11	8	0.578
Medication type				
SSRI	7	8	7	0.459
BDZ	1	4	1	
Education				
Under diploma	5	8	10	0.221
Diploma or higher	6	5	2	

*tDCS* transcranial Direct Current Stimulation, *M* mean, *SD* standard deviation; * = between-group differences in demographic variables were explored by Chi-square tests or Fisher’s exact test for categorical variables and *F* tests for continuous variables.

### Outcome measures (primary and secondary clinical measures, cognitive deficits, and brain physiology)

#### Primary and secondary clinical measures

The primary outcome measure to examine the effects of the intervention on OCD symptoms was the Yale-Brown Obsessive-Compulsive Scale (Y-BOCS) [43]. Additionally, anxiety and depressive states were tested by the Beck Anxiety Inventory (BAI) [44] and the Beck Depression Inventory (BDI-II) [45], respectively, and quality of life was assessed with the WHO Quality of Life Questionnaire (WHOQUL) [46]. These measures were used to evaluate the clinical efficacy of the intervention. The Y-BOCS is the most widely used clinician-rated interview for assessing OCD symptom severity and is a reliable measure of treatment-based reduction of symptoms [47]. The BAI is also well suited to monitor treatment outcomes [48], and the evaluated anxiety state is correlated with OCD symptoms [49, 50]. Similarly, BDI-II scores are associated with OCD symptoms [50], in line with the fact that around one-third of OCD patients suffer from comorbid depression [51]. A detailed description of these measures can be found in the supplementary information.

#### Cognitive assessment and brain physiology

We used a battery of neuropsychological tests that are sensitive to the cognitive deficit profile of OCD affected by interventions. Deficits of inhibitory control, working memory performance, and attention (e.g., sustained attention, set-shifting) are among the most well-documented cognitive deficits in OCD [37, 52, 53]. Importantly, these cognitive deficits are associated with frontal–striatal and frontal dysfunctions [29, 54–56], which are targeted by the intervention in this experiment. We examined response inhibition with the Go/No-Go task and Flanker test, working memory with the n-back task, and attention bias to OCD-related stimuli with an adapted dot-probe task. A detailed description of these measures is provided in the supplementary information. Finally, we monitored resting EEG to see how power spectrum and functional connectivity change after the intervention, specifically in frequency bands of interest (e.g., alpha, delta, gamma) [38–41]. A detailed description of the measures and EEG data preprocessing and analysis are in the supplementary content.

### tDCS

**Fig. 2:**
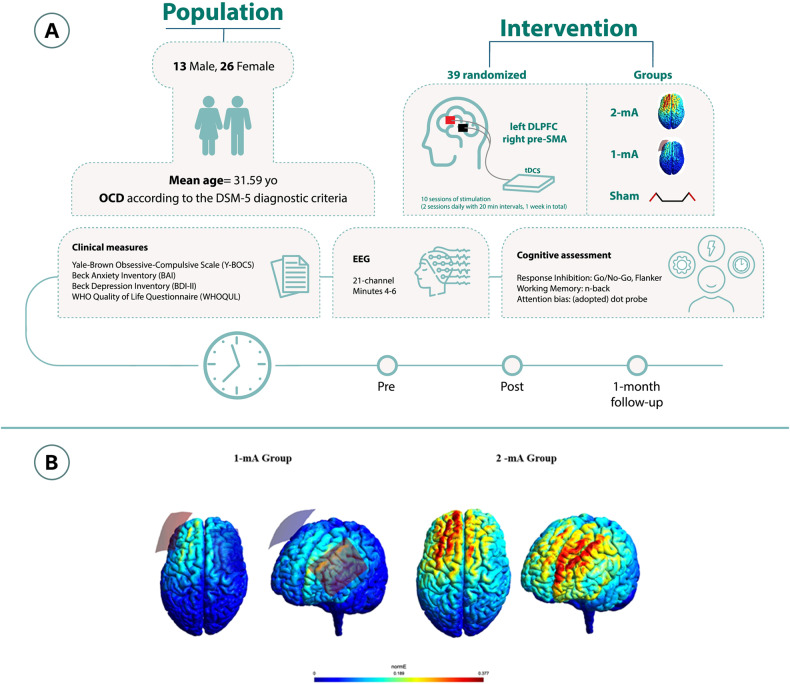
Study procedure and the intervention. **A** The experiment was conducted in a randomized, double-blind, sham-controlled parallel-group design. Participants were randomized to 3 tDCS arms: 1-mA tDCS (*n* = 12), 2-mA tDCS (*n* = 13), and sham tDCS (*n* = 12). **B** Results of the electrical field simulation for the current flow in the head based on the applied protocols. The anodal electrode (red) was placed over the left DLPFC and the cathode (blue) over the pre-SMA (FC2). The induced electric fields (EF) were calculated using SimNIBS [80], an open-source pipeline for Non-invasive Brain stimulation. (NIBS) modeling (available at https://simnibs.github.io/simnibs/). SimNIBS employs the mri2mesh tool, integrating FSL and FreeSurfer, to estimate the EF distribution. FSL segments extracerebral tissues, while FreeSurfer handles brain segmentation and gray matter surface reconstructions. Simulations were performed for the MNI-152 standard head [81]. Two stimulation protocols were modeled: one with 7 × 5 cm electrodes over the left DLPFC (F3) and the pre-SMA (FC2), with 1-mA current intensity, and the same parameters except for the intensity, which was 2-mA (10–20 EEG electrode positioning system). The average EF value (undirected) in each region was calculated based on the sum of EF values and the number of voxels in the area. All the calculations were performed using FSL and Matlab.

Direct currents were generated by an electrical stimulator (Oasis Pro, Mind Alive, Canada), and applied through a pair of saline-soaked sponge electrodes (7 × 5 cm) for two periods of 20 min and 20 min intervals between each stimulation period [36]. Stimulation was delivered on 5 consecutive days (two stimulations per day). In both active (1-mA, 2-mA) and sham conditions, anodal and cathodal electrodes were placed over the left DLPFC (F3), and right pre-SMA (FC2), respectively, to keep a minimum 6 cm distance between the edges of the electrodes [57]. To localize the right pre-SMA first, the pre-SMA was identified using the EEG 10–20 system for electrode positioning (i.e., 15% cm anterior to Cz) [17, 58]. In sham stimulation, the electrical current was ramped up and down for 30 seconds to generate the same sensation as in the active condition and then turned off [59]. To guarantee blinding, tDCS was applied by independent investigators who were not involved in outcome measures rating [60]. A side-effect survey was done after each tDCS session. Blinding efficacy was not explored among patients and experimenters. A 3D model of the current flow in the head was created to determine induced electrical fields in the brain for the above-mentioned tDCS protocol (Fig. 2).

### Procedure

Prior to the experiment, participants completed a brief questionnaire to evaluate their suitability for brain stimulation. All participants received 10 sessions of stimulation (2 sessions daily, 5 days in total) with 20-minute intervals between the sessions. To avoid confounding effects of the intervention at circadian non-preferred time, which can significantly affect neuroplasticity induction [61], all stimulation sessions took place between 11:00–14:00, and participants were not under sleep pressure [62]. Clinical cognitive measures were evaluated before the first intervention (pre-intervention), right after the end of the last intervention (post-intervention), and 1-month following the last stimulation session (follow-up). EEG measurements took place only before and after the intervention. Patients were instructed about the tasks before the beginning of the experiment. None of the patients received any kind of psychotherapy during the study. Participants were blind to the study hypotheses and stimulation conditions. The experimenter who conducted the outcome measures was blinded to the tDCS conditions (Fig. 2).

### Statistical analysis

Data analyses were conducted with the statistical package SPSS, version 26.0 (IBM, SPSS, Inc., Chicago, IL), and the GraphPad Prism 8.2.1 (GraphPad Software, San Diego, California). The normality and homogeneity of data distribution, and variance were confirmed by Shapiro-Wilk and Levin tests, respectively. Between-group differences in demographic variables were explored by Chi-square tests or Fisher’s exact test for categorical variables and F-tests for continuous variables. A multivariate Analysis of Variance (MANOVA) was first performed on the post-intervention and follow-up means of all outcome variables with group as the fixed factor and pre-intervention measures as covariates. This was to help protect against inflating the Type 1 error rate in the follow-up ANOVAs and post-hoc comparisons. A series of one-way ANOVA’s on each dependent variable was conducted as follow-up tests to the MANOVA. Finally, a series of post-hoc analyses were calculated using Dunnett’s multiple comparisons to examine individual mean difference comparisons across groups (active 1 mA, active 2 mA, sham) and time points (pre-intervention, post-intervention, follow-up). The critical level of significance was 0.05 for all statistical analyses.

## Results

### Side effects and baseline assessment

Participants tolerated the stimulation well, and no adverse effects were reported during and after stimulation, replicating the safety of the intervention [63, 64]. No significant difference was found between the group ratings of tDCS side effects (Supplementary Table S1). No significant between-group differences emerged in the pre-intervention measurements (Supplementary Table S2).

### Primary clinical outcome: reduction of OCD symptoms and anxiety

**Fig. 3:**
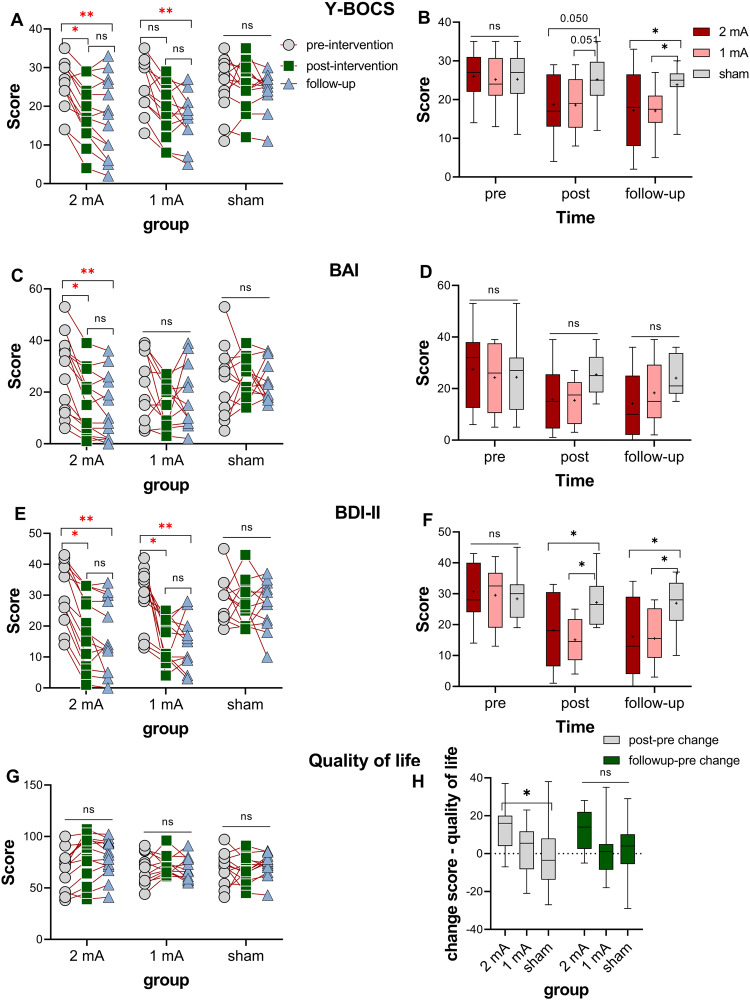
Clinical examination. OCD symptoms measured by Y-BOCS (**A**, **B**) and treatment-related variables (**C**–**H**) (anxiety measured by BAI, depressive state measured by BDI-II, and quality of life measured by the WHO quality of life scale), before and immediately after intervention and 1-month follow-up. Y-BOCS Yale-Brown Obsessive-Compulsive Scale, BAI Beck Anxiety Inventory, BDI-II Beck Depression Inventory-II. Floating asterisks [*] in the left panel represent a significant difference between pre-intervention measurement vs post-intervention and follow-up measurements in all groups. Floating asterisks [*] in the right-side figures indicate a significant difference between active stimulation groups (1 and 2-mA) vs sham tDCS at each time point. ns non-significant. All pairwise comparisons were conducted using Dunnett’s multiple comparisons. All error bars represent s.e.m.

A statistically significant MANOVA effect was seen for both post-intervention (Pillais’ Trace = 1.64, *F*_(24, 24)_ = 4.63, *p* < 0.001) and follow-up measurements (Pillais’ Trace = 2.98, *F*_(24, 24)_ = 4.63, *p* = 0.005). The results of the follow-up ANOVAs revealed a significant main effect of group on both Y-BOCS scores (post-intervention: *F*_(2, 22)_ = 7.14, *p* = 0.004, *ηp*^*2*^ = 0.394; follow-up: *F*_(2, 22)_ = 13.54, *p* < 0.004, *ηp*^*2*^ = 0.552) and BAI scores (post-intervention*: F*_(2, 22)_ = 8.78, *p* = 0.002, *ηp*^*2*^ = 0.423; follow-up: *F*_(2, 22)_ = 5.78, *p* = 0.010, *ηp*^*2*^ = 0.345). Next, Dunnett’s multiple comparisons were performed on individual mean difference and showed a significant decrease in Y-BOCS scores at the post-intervention time in the 2-mA group (*p* = 0.021, *d* = 0.98), at the 1-month follow-up in both the 2-mA (*p* = 0.004, *d* = 1.01) and 1-mA (*p* = 0.013, *d* = 1.23) groups as compared to pre-intervention time, but no significant changes were seen in the sham group (Fig. 3A). When compared to the sham group, reduced Y-BOCS scores were significant in both active groups only at the follow-up (1-mA: *p* = 0.044, *d* = 1.16; 2-mA: *p* = 0.045, *d* = 0.82) (Fig. 3B). For the BAI scores, Dunnett’s multiple comparisons showed a significant decrease in BAI scores from pre-intervention to both post-intervention (*p* = 0.025, *d* = 0.85) and 1-month follow-up (*p* = 0.009, *d* = 0.97) only in the 2-mA group (Fig. 3C). When compared to the sham group, both active groups showed a non-significant trendwise reduction of BAI scores at the post-intervention assessment (1-mA: *p* = 0.074; 2-mA: *p* = 0.083) (Fig. 3D).

### Secondary clinical outcomes: mood and quality of life

The results of the follow-up ANOVAs revealed a significant main effect of group on BDI-II scores (post-intervention: *F*_(2, 22)_ = 7.13, *p* = 0.004, *ηp*^*2*^ = 0.394; follow-up: *F*_(2, 22)_ = 10.47, *p* < 0.001, *ηp*^*2*^ = 0.488) and quality of life (post-intervention: *F*_(2, 22)_ = 3.58, *p* = 0.045, *ηp*^*2*^ = 0.246; follow-up: *F*_(2, 22)_ = 7.33, *p* = 0.004, *ηp*^*2*^ = 0.400). Dunnett’s post hoc tests showed that BDI-II scores were reduced from the pre-intervention to both post-intervention and 1-month follow-up assessment in both, 2-mA (post-intervention: *p* = 0.002, *d* = 1.15; follow-up: *p* < 0.001, *d* = 1.34) and 1-mA (post-intervention: *p* < 0.001, *d* = 1.65; follow-up: *p* = 0.001, *d* = 1.49) groups, but not in the sham group, and reduced depressive state at each time point was significant in both active groups vs. the sham group (Fig. 3E, F). No significant individual mean differences were found across groups in quality-of-life scores (Fig. 3G). However, we calculated the changes in quality of life scores from the baseline to post-intervention and follow-up. Dunnett’s post hoc test of score changes across groups showed that quality of life scores significantly improved after the intervention only in the 2 mA group (*p* = 0.025) (Fig. 3H).

### Cognitive functions: improved inhibitory control in both active tDCS groups

**Fig. 4:**
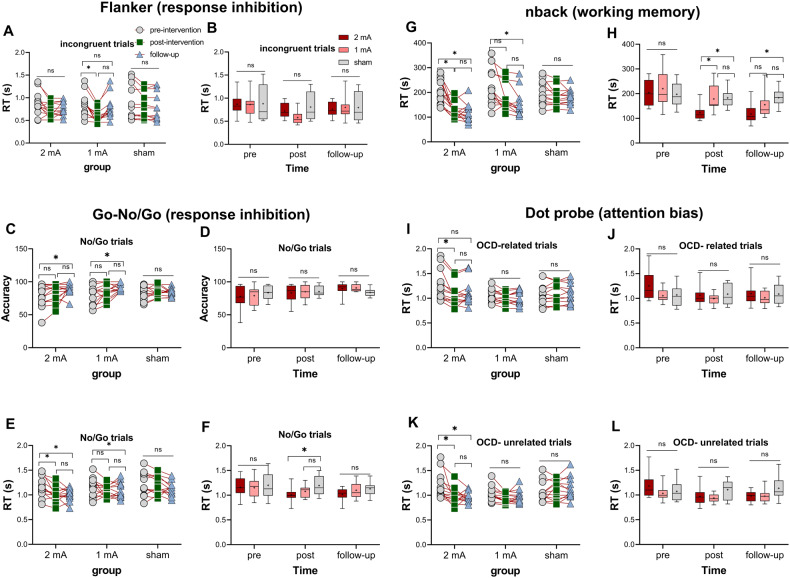
Neuropsychological performance. Response inhibition measured by Flanker (**A**, **B**) and Go-No/Go (**C**–**F**) tasks, before, immediately after the intervention, and at the 1-month follow-up. Working memory and attention bias were measured by n-back (**G**, **H**) and dot-probe (**I**–**L**) tasks, before, immediately after the intervention, and 1-month follow-up. RT Reaction time, s seconds. Floating asterisks [*] in the left panel represent a significant difference between pre-intervention measurement vs post-intervention and follow-up measurements in all groups. Floating asterisks [*] in the right panel indicate a significant difference between active stimulation groups (1 and 2-mA) vs sham tDCS at each time point. ns non-significant. All pairwise comparisons were conducted using Dunnett’s multiple comparisons. All error bars represent s.e.m.

In the Flanker test, the follow-up ANOVAs revealed a significant main effect of group on both, congruent (post-intervention: *F*_(2, 22)_ = 10.08, *p* < 0.001, *ηp*^*2*^ = 0.478; follow-up: *p* = 0.901) and incongruent trials (post-intervention: *F*_(2, 22)_ = 8.01, *p* = 0.002, *ηp*^*2*^ = 0.422; follow-up: *p* = 0.445) only after the intervention and not follow-up. Dunnett’s multiple-test comparisons revealed a significant *pre* vs *post*-intervention RT reduction of incongruent stimuli (*p* = 0.015, *d* = 1.35) only in the 1-mA group, which, however, was not significant vs the sham (Fig. 4A, B). In the Go/No-Go task, the results of the follow-up ANOVAs revealed a significant main effect of group for No-Go trials reaction time (post-intervention: *F*_(2, 22)_ = 7.11, *p* = 0.004, *ηp*^*2*^ = 0.393; follow-up: *F*_(2, 22)_ = 4.34, *p* = 0.026, *ηp*^*2*^ = 0.283) and a marginally significant effect on No-Go trials accuracy at the post-intervention measurement (*F*_(2, 22)_ = 3.39, *p*_follow-up_ = 0.054, *ηp*^*2*^ = 0.233). Dunnett’s multiple-test comparisons showed increased accuracy from the pre-intervention to the follow-up measurement in 2-mA (*p* = 0.022, *d* = 0.87) and 1-mA (*p* = 0.032, *d* = 0.27) groups (Fig. 4C). The 2-mA protocol significantly reduced RT from pre vs post-intervention (*p* = 0.027, *d* = 1.92) and pre vs follow-up (*p* = 0.037, *d* = 1.95) as well (Fig. 4E) and here the performance speed on the No-Go trials was significantly faster in the 2-mA group vs the sham after the intervention (*p* = 0.014, *d* = 1.73) (Fig. 4F).

### Cognitive functions: working memory and attention bias

In working memory performance, the follow-up ANOVAs revealed a significant main effect of group only on performance speed after the intervention (*F*_(2, 22)_ = 16.76, *p* < 0.001, *ηp*^*2*^ = 0.604) and 1-month follow-up (*F*_(2, 22)_ = 13.87, *p* < 0.001, *ηp*^*2*^ = 0.558). Dunnett’s multiple comparisons showed a significantly faster pre- vs post-intervention RT (*p* < 0.001, *d* = 1.92) and pre vs follow-up RT (*p* < 0.001, *d* = 1.95) in the 2-mA and a significant pre vs follow-up RT reduction (*p* = 0.003, *d* = 1.01) in the 1-mA group. In the 2-mA group, this RT reduction was furthermore larger than that of the sham group at post-intervention (*p* = 0.007, *d* = 1.73) and follow-up (*p* = 0.002, *d* = 1.91) measurements (Fig. 4G, H). Finally, in the attention bias of patients to OCD-related stimuli, the follow-up ANOVAs showed a significant main effect of group for both, OCD-related (post-intervention: *F*_(2, 22)_ = 8.82, *p* = 0.002, *ηp*^*2*^ = 0.445; follow-up: *F*_(2, 22)_ = 5.53, *p* = 0.011, *ηp*^*2*^ = 0.335) and unrelated stimuli (post-intervention: *F*_(2, 22)_ = 5.82, *p* = 0.009, *ηp*^*2*^ = 0.346; follow-up: *F*_(2, 22)_ = 9.45, *p* = 0.001, *ηp*^*2*^ = 0.462). Dunnett’s multiple-test comparisons showed a significantly faster pre- vs post-intervention RT for both, OCD-related (*p* < 0.026, *d* = 0.70) and unrelated (*p* < 0.007, *d* = 0.93) stimuli in the 2-mA group (Fig. 4I, K). No significant between-group RT differences were, however found for the post-intervention and follow-up measurements.

### Intervention-related changes in EEG power spectrum density and functional connectivity

**Fig. 5:**
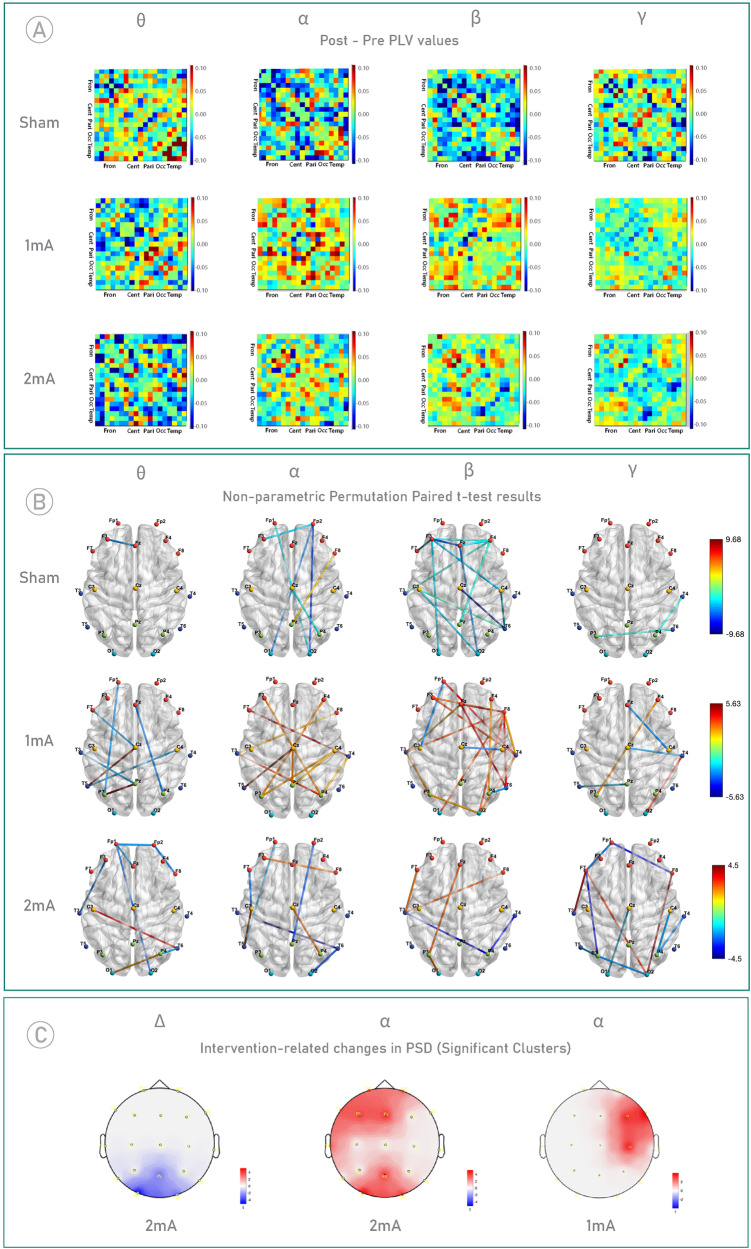
Functional connectivity and Power spectrum density (PSD) analyses. **A** Post-minus-pre tDCS intervention connectivity matrices (PLV) averaged across participants in each intervention group for theta, alpha, beta, and gamma frequencies. Only the first four letters of the respective scalp regions are used for an abbreviation: Frontal (Fron), Central (Cent), Parietal (Pari), Occipital (Occ), Temporal (Temp). **B** Within-group intervention-related changes in PLV. Visualization of the edge-level data with significant subnetworks in each frequency band. The size and color of the edges are determined by the t-value. Red edges represent an increase in functional connectivity between respective nodes, and blue edges represent a decrease in functional connectivity between respective nodes. Node colors represent specific regions- Frontal (Red), Central (Yellow), Parietal (Green), Occipital (Cyan), and Temporal (Dark Blue). Associated electrodes within each region are presented in supplementary materials. **C** Intervention-related changes in PSD. A cluster-based permutation test was conducted on relative PSD values (post-intervention minus pre-intervention) within each active group in comparison to the sham group, identifying significant clusters. The left panel represents a statistically significant decrease in relative delta power in the 2-mA group compared to the sham group (cluster-level statistic = −11, *p* < 0.05). The middle panel illustrates an increase in relative alpha power in the 2-mA group compared to the sham group in the left frontal region (cluster-level statistic = 16, *p* < 0.01), and occipital region (cluster-level statistic = 11, *p* < 0.05). The right panel displays a marked increase in relative alpha power in the 1-mA group relative to the sham group, with a significant cluster identified in the right frontal region (cluster-level statistic = 11, *p* < 0.05).

PSD Analysis with a cluster-based permutation test (post vs pre) revealed a significant increase in relative alpha power in the left frontal region (cluster-level statistic = 16, *p* < 0.01) and the occipital region (cluster-level statistic = 11, *p* < 0.05) in the 2-mA group compared to the sham group. Additionally, a significant decrease in relative delta power was observed in a cluster located in the occipital region (cluster-level statistic = −11, *p* < 0.05) in the 2-mA group compared to the sham group. In the 1-mA group, a significant increase in relative alpha power was observed in the right frontal region (cluster-level statistic = 11, *p* < 0.001) (Fig. 5C). Topographical plots of the relative power changes (post power – pre power) in all frequency bands after each intervention are in supplementary information (Fig. S1). Regarding functional connectivity, comparative analysis of post-intervention Phase Locking Value (PLV) matrices showed a general trend of increased connectivity in higher frequency bands in both active groups as compared to the sham group (Fig. 5A, B and Fig. S2). When we compared both groups with each other, the 2-mA group generally decreased functional connectivity across most frequency bands (EEG connectivity results are fully described in the supplementary information). We did not see any relevant correlation between EEG parameters and clinical/cognitive measures.

## Discussion

In this randomized, double-blind, sham-controlled, parallel-group clinical trial, we investigated the impact of an intensified tDCS protocol (stimulation twice per day with 20-min intervals) over the prefrontal-supplementary motor network, in two dosages (1-mA vs. 2-mA) on primary clinical symptoms, neuropsychological performance, and electrophysiological correlates in patients with OCD. The 2-mA stimulation dosage significantly reduced OCD symptoms and anxiety after the intervention and in the follow-up. Both active stimulation protocols significantly reduced depressive symptoms. At the neuropsychological level, both active protocols partially improved response inhibition, and the 2-mA protocol reduced attention bias to threat-related stimuli and improved working memory performance as well. Both protocols increased alpha, and the 2-mA protocol decreased delta oscillatory power too. Both protocols increased connectivity in higher frequency bands at frontal-central areas compared to the sham. No significant changes were observed in the sham group for any outcome measures.

These findings can be explained from neurophysiological and neuropsychological perspectives. The hallmark finding of neuroimaging studies refers to lateral hypoconnectivity (including the DLPFC) and medial hyperconnectivity (including the pre-SMA) in OCD [37, 65], which was the rationale for applying our stimulation protocol and is in line with findings from rTMS studies [66]. We applied anodal stimulation over the left DLPFC to increase the activity of this region and cathodal stimulation of the pre-SMA to downregulate activity. With causal modulation of cerebral excitability with tDCS [6], we expected to restore functional abnormalities in the OCD-relevant brain circuitry, and in principal accordance, this intervention was associated with behavioral and clinical improvement in this study. In further accordance, the intervention, especially after 2-mA stimulation, restored altered alpha and delta oscillatory power in patients [38], and both protocols increased connectivity in the prefrontal regions, which is reduced in OCD patients, that can be likely an appropriate treatment cortical target [21, 66].

In addition to neurophysiological changes, neuropsychological accounts could also explain our findings. The most well-known psychological mechanisms underlying OCD psychopathology include impaired cognitive control (the inability to regulate compulsive behavior) [67], impaired cognitive flexibility (the inability to regulate thinking) [68], and impaired balance between goal-directed behavior and more automatic habit learning [69, 70]. Importantly, these cognitive abilities are related to lateral and medial prefrontal cortices [23, 37, 71]. The behavioral tasks we used are primarily related to cognitive control and cognitive flexibility (e.g., response inhibition, working memory), and the performance of these tasks was significantly improved after intervention, more obviously in the 2-mA group. Here, it should however be noted that the effects of both protocols on response inhibition were smaller than expected, which could be due to the higher relevance of the right prefrontal region in cognitive inhibition [72]. That said, anodal stimulation of the left DLPFC was also shown to improve executive functions in neuropsychiatric patients in previous studies [13, 36, 73, 74], and might explain treatment effects in OCD patients.

One major rationale of this study was to identify the effect of different stimulation dosages on treatment efficacy, specifically in the intensified protocol, which we had already applied in another study with promising results [36]. This protocol has not been applied in OCD to the best of our knowledge. Our results in this study show that the 2-mA intensified tDCS protocol was overall more effective than both, sham stimulation and the 1-mA stimulation, especially for the clinical variables, and it improved more outcome measures including measures of behavioral performance, compared to the 1-mA protocol (e.g., working memory, attention bias). The rationale behind the protocol comes from a study showing that twice-stimulation with 20-minute intervals leads to longer aftereffects on cortical excitability compared to non-repeated stimulation or stimulation with long intervals and resembles features of late-phase LTP [35, 75]. This finding has at least two important clinical implications. First, the 2-mA stimulation is associated with higher clinical efficacy in OCD, and probably in other anxiety disorders, as shown in our previous work in patients with social anxiety disorder [36]. Second, the intensified stimulation (twice per day with a 20 min interval), has significant clinical efficacy for treatment-related variables. This is in line with physiological studies that have shown that repeated tDCS sessions induce larger increases in excitability [76] and more importantly suggest that the intensified protocol (repetition of two 20-minute stimulation with a 20-minute interval between) can be promising for clinical application in other neuropsychiatric diosders.

Our protocol was different from other commonly applied protocols in other aspects. First and to the best of our knowledge, none of the previous tDCS randomized trials targeted the prefrontal-SMA network by stimulating both left DLPFC and pre-SMA [77, 78]. Additionally, this is also the first randomized-controlled trial that compared the efficacy of two stimulation dosages which is typically needed for establishing clinical efficacy of an intervention. Finally, in comparison to other protocols used in previous studies, a recent metanalysis of tDCS RCTs in OCD showed that protocols that applied cathodal stimulation over the pre-SMA with an extracephalic return electrode delivered stronger electric fields to the circuity involved in OCD in comparison to the other montages [19]. None of these tDCS studies targeted the left DLPFC with anodal tDCS. This metanalysis, however, did not find significant differences between active vs sham tDCS in contrast to our study.

Our study had several limitations. First, the intrinsically limited focality of tDCS can result in a relatively diffuse stimulation. Neuroimaging methods can help to more accurately identify the regions directly affected by tDCS in future studies. Furthermore, we did not examine blinding efficacy in patients and could not measure EEG in the follow-up due to COVID-19-related restrictions. With respect to blinding efficacy, the 2-mA intensity typically results in more sensations over the skin as compared to the sham and 1-mA protocol, which may affect patients’ blinding. However, there was no significant difference in reported ratings of tDCS side effects between groups (see supplementary information, Table S1).

Taken together, our findings suggest that the intensified prefrontal-supplementary motor cortex tDCS protocol introduced for the treatment of OCD is promising and might be effective in other neuropsychiatric disorders. Both primary OCD symptoms and secondary treatment-related variables (anxiety, depressive state, quality of life) and cognitive functions (response inhibition, working memory, and attentional bias) improved after the intervention, especially in the 2-mA group. Partial effects of the intervention on response inhibition might suggest further optimizing the protocol by targeting the right prefrontal cortex, which was not the primary target here, and the sessions were relatively low. Both protocols also significantly restored brain oscillatory power in frequency bands introduced as biomarkers of OCD. In line with rTMS intervention [8, 79], tDCS may also hold the potential to serve as a therapeutic intervention in OCD treatment. Future larger trials with longer follow-up assessments are needed to support the clinical efficacy of this intervention.

### Supplementary information

Supplementary information

Figure S1

Figure S2
