# Supplementary material for: Targeting the prefrontal-supplementary motor network in obsessive-compulsive disorder with intensified electrical stimulation in two dosages: a randomized, controlled trial
Source: Transl Psychiatry. 2024 Feb 5;14:78. doi: 10.1038/s41398-024-02736-y (PMC10844238; doi:10.1038/s41398-024-02736-y)
Supplement: Supplementary file 1 — Supplementary information [file 41398_2024_2736_MOESM1_ESM.docx]

**Supplementary information**

1. ***Randomization and blinding***

A random allocation sequence table was generated by an online block randomization website (<https://www.sealedenvelope.com/simple-randomiser/v1/lists> ) for three treatment groups and a block size of 6. The allocation of participants was concealed with the unique generated code for each patient and sealed in opaque envelopes, prepared prior to the study by a research assistant, who was not involved in the study. The intervention was applied by separate investigators who were not involved in the assessment of outcome measures and the experimenter who conducted the outcome measures was blinded to the tDCS conditions.

1. ***Descriptions clinical measures***

*Yale-Brown Obsessive-Compulsive Scale (Y-BOCS):* The Y-BOCS is the most widely used clinician-rated interview for assessing OCD symptom severity with adequate psychometric characteristics (i.e., inter-rater reliability and predictive validity) (Maust et al., 2012). It contains 10 items, and each item is rated from 0 (no symptoms) to 4 (extreme symptoms). The Y-BOCS is sensitive to change, and during-treatment score reductions are valid outcome indicators (Maust et al., 2012). Therefore, the results of this questionnaire are suited as clinical predictors of treatment response, as shown by previous rTMS studies (Rostami et al., 2017). The Y-BOCS items weigh obsessions and compulsions equally. Obsession items assess spent time on obsessions (item 1), interference (item 2) and distress (item 3) due to obsessive thoughts, resistance against obsessions (item 4) and degree of control over obsessive thoughts (item 5). Items 6-10 assess respective variables (i.e., spent time, interference, distress, resistance, and degree of control) for compulsions respectively.

*Beck Anxiety Inventory (BAI)*: The BAI consists of 21 items, which are rated on a Likert scale ranging from 0 to 3, resulting in raw scores ranging from 0 to 63, and are indicative of the presence of anxiety. The BAI is well suited to monitor anxiety treatment outcomes (Leyfer et al., 2006), and the obtained anxiety state is correlated with OCD symptoms (Velloso et al., 2016; Reuman et al., 2017).

*Beck Depression Inventory-II (**BDI-II)*: The BDI-II (Beck et al., 1996) is a 21-item self-report inventory about how the subject has been feeling in the last two weeks. The internal correlational coefficient of the BDI-II is reported between 0.7- 0.92 and the test-retest reliability coefficient is reported 0.93 at the one-week interval (Beck et al., 1988). A native language version of the BDI-II with adequate psychometric properties was used in this study (Ghassemzadeh et al., 2005). A score of 20–28 and higher in the BDI-II is indicative of moderate to severe depression. A Cronbach's alpha of 0.94 is reported for the Persian language version of the BDI-II (Alipoor and Nori, 2006).

*Quality of life:* Quality of life domains were assessed with the short version of the World Health Organization Quality of Life (WHOQUL) questionnaire(Skevington et al., 2004). The questionnaire is a cultural-independent measure of the quality of life and assesses the quality of life in four domains: physical, psychological, social and environmental health. It has 26 items and each item is rated on a 5-point scale with a higher score indicative of higher quality of life. The Cronbach's alpha of physical, psychological, social and environmental health are reported 0.80, 0.76, 0.66 and 0.80 respectively (Skevington et al., 2004). The reported Cronbach's alpha of the Persian version is reported higher than 0.7 in all domains and for the whole scale is 0.73 (Nejat et al., 2006).

1. ***Neuropsychological battery***

*Flanker test*

This task measures the ability to inhibit the response to irrelevant, interfering, and rival stimuli (Eriksen and Eriksen, 1974). In interference control, inadequate or irrelevant part(s) of information are filtered to improve selective attention to adequate and relevant part(s) of information. This type of inhibitory control is measured by the Stroop (Simon and Berbaum, 1990), and flanker (Eriksen and Eriksen, 1974) tasks. In these tasks, individuals are instructed to selectively attend to a stimulus and/ or response in the presence of other irrelevant stimuli and/ or responses, which should be ignored. In the present study, an arrow version of the flanker task was used. In this version of the task, an arrow, pointing to the right or left, appears in the center of a screen, and the participant is instructed to press the corresponding arrow key on a keyboard as fast and accurately as possible. The target stimulus is surrounded by two pairs of distractors on the right and left sides. The task has three stages based on the type of distractor, namely congruent, incongruent, and neutral. Each stage consists of 50 trials that are presented in a mixed pseudorandom order. In each trial, after a fixation cross (1 s), the stimuli appear shortly (500 ms), and responses have to be made within 1 s after presentation. The primary outcome of the task is accuracy. This task is suitable for children from 5 years of age (Ridderinkhof et al., 1997). The outcome measures of this task are the reaction time of congruent, incongruent and neutral stages and the flanker task index as the main outcome measure, which is calculated as reaction time in the congruent minus the incongruent stages.

*Go/No-Go task*

To measure response inhibition, we also used the Go/No-Go task, which is widely used as a measure of response inhibition and involves activation of prefrontal regions, mainly the DLPFC, orbitofrontal cortex and inferior frontal gyrus (Criaud and Boulinguez, 2013). Participants are exposed to stimuli in a continuous stream and need to make a binary decision on each stimulus by pressing a button (Go) for a specific stimulus and not pressing the same button (No-Go) for a different stimulus. In this study, participants were presented with the picture of a plane, which appeared on the screen in four directions (up, down, left, and right). They were instructed to press the button aligned with the plane presented in any direction (the Go condition) but had to withhold pressing any button when a sound (“Beep”) was provided (the No-Go condition). This task consisted of 50 stimuli that required response execution in 75% of trials, and inhibition of a response in the remaining 25%. The planes (7 × 7 cm) were static and black and were presented on a white screen. Major outcome variables include accuracy of No-Go and Go responses and the response time, which is the average time participants spend to press the button. The latter examines prepotent response inhibition as an index of inhibitory control. This task takes about 7 minutes to complete.

*Working memory*

In order to assess Working memory performance, we used the visual “*n*-back” task which is a standard “executive” WM task (Kane et al., 2007) involving the PFC (Owen et al., 2005). In the *n*-back task, participants are exposed to stimuli (letters or pictures, one stimulus per time-point) presented on a screen. The task requires to identify the pictures identical to the ones presented in items before. We applied the 1-back test, in which the target was any picture that was identical to the one it preceded. Participants were instructed to press “1” in case of identical pictures (i.e., hits), and “2” in case of different pictures on a keyboard. Participants had thus to respond to all stimuli. This required online control over all stimuli and constant updating of information. The task included 100 stimuli in total, which were composed of 10 different images. Each image was randomly repeated 3 times in each run (Fig 1). We used accuracy and mean reaction time (RT) of correct responses as measures of WM performance.

*Dote probe*

Attention bias to threat stimuli was measured using the dot-probe paradigm (MacLeod et al., 1986). Previous studies have shown that OCD patients have attention bias toward OCD-related stimuli (e.g. washing- and checking-relevant stimuli) (van den Heuvel et al., 2005; Moritz et al., 2009). Two stimuli are presented to the participants that appear randomly on either side of the screen for a pre-determined time before an asterisk is presented at the location of one former stimulus. Participants are instructed to indicate the location of the asterisk as quickly as possible via the response box. In this computerized task, 80 words (40 OCD related, 40 neutral) were selected. 40 words represented OCD-related obsession (washing- and checking-relevant stimuli) and 40 ones represented neutral words. Stimuli of each condition (OCD vs neutral) were presented in each measurement in random order. During the task, a fixation cross was shown initially in the center of the screen for 1000 ms. A pair of two words (OCD-related/neutral, neutral/neutral) were then presented simultaneously on the left and right side of the screen from 500 ms. Immediately following the presentation of words, a dot probe was presented at the location of one of the words (OCD-related vs neutral). Participants were asked to indicate the location of the probe by pressing the corresponding key as fast as possible. Their reaction time served as a measurement of selective attention. Stimuli presentation was controlled by a laptop with a 15.2” screen (Schneider et al., 2002), at a viewing distance of approximately 50 cm.

1. ***Side effects of tDCS***

All participants tolerated tDCS well and no adverse effects were reported during and after stimulation, except for a mild itching, tingling, and burning sensation under the electrodes during approximately the first 30 s of stimulation in each tDCS condition. The occurrence of side effects is summarized in Table S1. Each side effect was rated on a 0-4 Likert-type scale with 0 representative of no sensation and 4 indicative of extreme sensation. Moreover, the perceived side effects and evaluations after each tDCS session were not significantly different between stimulation conditions (See Table S1).

1. ***EEG preprocessing and data analyses***

*4.1. EEG Preprocessing and Artifact Removal*

EEG signals were recorded with a 21-channel EEG device (SD-C24, Shole Danesh Co, Tehran, Iran), and the included electrodes were Fz, Cz, Pz, C3, T3, C4, T4, Fp1, Fp2, F3, F4, F7, F8, P3, P4, T5, T6, O1, O2, A1, A2. To preprocess and remove artifacts from the EEG data, we used Makoto's preprocessing pipeline in the EEGLAB toolbox 2022.1 (Delorme and Makeig, 2004) with MATLAB 2022b (The MathWorks, Natick, MA). The data were first resampled to 512 Hz, high-pass filtered at 1 Hz, and re-referenced to an average reference. We used the CleanlineNoise plugin in EEGLAB for line noise removal. We then applied ASR (Artifact Subspace Reconstruction), an automated algorithm that eliminates flatline and noisy channels, low-frequency drifts, and short-time bursts. Any removed channels were interpolated using the spherical interpolation method. Afterward, we visually inspected all the raw data to detect artifact-related parts. To remove non-brain artifacts, we applied Adaptive Mixture ICA (AMICA) to the EEG data to decompose independent components (ICs). We then used the EEGLAB plugin ICLabel to identify brain ICs (with a 'brain' label probability of more than 0.8) from all types of ICs, including Brain, Muscle, Eye, Heart, and others. Finally, we extracted a 60-second segment from the middle part of each preprocessed EEG and exported it to the BRAINSTORM software (version November 2022) for further analysis (Tadel et al., 2011).

*4.2. Power Spectral Density analysis*

To compute PSD (Power Spectral Density), we used Welch's method (Welch, 1967) on 60-second segments of each condition, with 4-second sliding Hamming windows overlapping at 50%. PSD is a measure of the power of a signal at different frequencies and is commonly used in EEG analysis to identify frequency bands associated with different cognitive processes. We used the (Niso et al., 2019) pipeline with BRAINSTORM software to perform the analysis. We grouped the PSD values into typical frequency bands of EEG: Theta (5-7 Hz), Alpha (8-12 Hz), Beta (15-29 Hz), and Gamma (30-45 Hz). We computed the PSD absolute values at each frequency bin relative to the total power across the entire frequency spectrum. This allowed us to standardize the PSD values and compute the relative power for each frequency band. We then obtained relative power values for each electrode, which were used for statistical analysis. Topographical plots of the relative power changes are shown in Fig. S1.

*4.3. Functional connectivity analysis*

Functional connectivity on the scalp was assessed using Phase Locking Value (PLV) (Lachaux et al., 1999), a method that quantifies phase relationships between pairs of channels or sources. PLV values range from 0 to 1 and provide a measure of the degree of phase synchronization between two signals. To compute the PLV, we filtered each 60-second segment of the time series into four frequency bands - Theta (5-7 Hz), Alpha (8-12 Hz), Beta (15-29 Hz), and Gamma (30-45 Hz) - using a finite impulse response (FIR) bandpass filter. We obtained the instantaneous phase of each filtered signal using the Hilbert transform and then calculated the PLV for each frequency band to obtain a connectivity matrix for each condition. Each connectivity matrix consisted of 19 x 19 elements, reflecting the 19 channels used for EEG analysis. We performed all of these steps using the BRAINSTORM software.

- 1. *EEG changes: Intervention-related changes in functional connectivity*
     1. *Comparison of Pre- Vs Post Intervention functional connectivity within-groups*

Utilizing a non-parametric permutation paired t-test on the Post Vs Pre condition PLV matrices within each groups, we identified notable differences in subnetworks in every frequency band. These differences, significant after FDR correction (*p*≤0.05), were characterized by both increased and decreased functional connectivity across the frequency bands.

In the 2-mA group, the C3-T6 electrodes demonstrated the most pronounced increase in connectivity (t= 3.58, *p*=0.003) within the theta band. Conversely, the Fp1-O2 electrodes recorded the largest decrease (t= -2.67, *p*=0.022). Within the alpha band, substantial increases in connectivity were observed in frontal and centro-temporal regions, specifically at C3-T5 (t= 2.5, *p=*0.036) and F3-F8 (t= 2.3, *p*=0.041) electrodes. Conversely, the T3-T6 electrodes in the centro-temporal region displayed the most substantial decrease in connectivity (t= -5.04, *p=*0.001). In the beta band, fronto-temporal regions displayed the greatest connectivity increase, especially the T3-F8 (t= 2.44, *p=*0.034) and T3-F7 (t= 2.38, *p=*0.047) electrodes. Conversely, the most prominent decrease in connectivity within this band was in the temporo-parietal region, especially at electrodes P4-T3 (t= -3.61, *p=*0.001) and P4-T4 (t= -3.51, *p =* 0.005). Lastly, in the gamma band, the greatest enhancements in connectivity were located in the fronto-temporal regions, with the F7-F3 (t= 4.16, *p*=0.004) and F8-O2 (t= 4.5, *p* = 0.006) electrodes showing the largest increases. Meanwhile, the most considerable decreases in connectivity were identified in the frontal and fronto-parietal regions, specifically at the F7-P3 (t= -3.87, *p*=0.003) and Fp1-F8 (t= -3.36, *p*=0.004) electrodes (Fig 6-B).

The 1-mA group presented similar trends. In the theta band, the P3-Pz (t= 5.63, *p*=0.006) and Cz-T5 (t= 3.82, *p*=0.010) electrodes, located in the parietal and centro-temporal regions, displayed the largest increases in connectivity. On the other hand, the frontal-parietal region, particularly the Fz-P4 (t= -2.94, *p*=0.015) and Fp1-P3 (t= -2.70, *p*=0.027) electrodes, showed the greatest decrease. In the alpha band, we noticed a widespread increase in connectivity spanning various regions, with the most notable enhancements in the fronto-temporal regions, specifically at the F7-T4 electrodes (t= 5.35, *p*=0.007). In the beta band, the majority of regions under various electrodes showed an overall increase in connectivity. Notably, the largest increases were found in the frontal and fronto-temporal regions, particularly at the Fz-F8 (t= 5.49, *p=*0.035) and F8-T6 (t= 4.58, *p=*0.035) electrodes. The most pronounced decrease, however, occurred in the central region, specifically at the C4-Cz electrodes (t= -3.17, *p*=0.011). Lastly, in the gamma band, the T4-O2 electrodes (t= 3.80, *p*=0.011) showed the greatest increase in connectivity, while the largest decrease was detected at the Fz-C4 electrodes (t= -2.74, *p*=0.035) (Fig 6-B).

In the sham group, a general trend towards decreased functional connectivity across all frequency bands was observed, with the alpha and beta frequencies showing the most substantial reductions. An exception to this trend was seen in the alpha band, where an increase in connectivity was noted at the F8-Pz electrodes. However, the largest reductions in connectivity were identified at the Fp2-O2 (t= -6.34, *p*=0.030) and Fp1-O1 (t= -4.92, *p*=0.030) electrodes within the same band. In the beta band, the majority of monitored regions demonstrated a decrease in connectivity, with the T6-Cz electrodes showing the most marked decrease (t= -9.68, *p*=0.030) (Fig 6-B).

- - 1. *Comparison of Post-Intervention functional connectivity*

Using a non-parametric permutation independent t-test on the post-intervention Phase Locking Value (PLV) matrices between distinct experimental groups, we discerned substantial disparities in subnetworks across all frequency bands (FDR corrected *p*≤0.05).

When comparing the 2-mA group's post-intervention functional connectivity with that of the sham group, we observed a general trend of decreased functional connectivity (as evident by PLV values) in the theta frequency and an overall trend of increased functional connectivity in higher frequency bands in the 2-mA group compared with the sham group. In the theta band, the most substantial reductions in PLV values were found primarily in fronto-central regions, particularly in F7-C4 (t= -3.64, *p*=0.005) and Fp2-C3 (t= -3.10, *p*=0.010). In the alpha band, the most prominent increase in functional connectivity compared to the sham group was evident in fronto-central regions, particularly Fz-C3 (t= 2.82, *p*=0.008) and Fp1-C4 (t= 2.80, *p*=0.015), while a decrease was detected at the F4-F7 electrodes (t=-2.66, *p*=0.021). In the beta band, there was a general trend toward increased functional connectivity in the 2-mA group compared to the sham group, especially in frontal and fronto-central regions, with the most substantial increases in Fz-C3 (t=2.95, *p*=0.015) and F4-Fp1 (t=3.00, *p*=0.013). In the gamma band, the greatest enhancements were observed in the F4-T3 electrodes (t= 4.37, *p*=0.002), while the most substantial decrease was detected at T3-O1 (t= -3.17, *p*=0.009) (Statistical results in Fig. S2).

Comparing the 1-mA group with the sham group, we observed a similar pattern of decreased functional connectivity in the theta frequency and a general trend of increased functional connectivity in higher frequency bands as we saw in 2-mA group. In the theta band, reductions in PLV values were mainly detected in fronto-central and frontal regions, such as at Fp1-Cz (t= -4.22, *p*=0.002) and F7-C3 (t= -3.15, *p*=0.009), whereas enhanced connectivity was observed in fronto-temporal regions, including Fp1-T6 (t=4.02, *p*=0.002) and Fp1-T5 (t= 3.29, p= 0.012). For the alpha band, a general enhancement of functional connectivity was apparent in the 1-mA group compared to sham across various regions, with notable increases in temporal and fronto-temporal regions such as T6-T4 (t= 4.18, *p*=0.003) and Fz-T3 (t= 3.14, *p*=0.005). In the beta band, increased connectivity was generally observed in the 1-mA group, with T3-Fp2 (t= 7.16, *p<*0.001) showing the highest functional connectivity. Likewise, in the gamma band, augmented functional connectivity was identified across numerous regions, with notable increases at C4-P4 (t= 4.32, *p*=0.002) and F4-T3 (t= 4.63, *p<*0.001) (Fig. S2).

When comparing the 2-mA group to the 1-mA group in the post-intervention period, we observed predominantly lower functional connectivity in the 2-mA group. In the theta band, the 2-mA group generally displayed lower PLV values across most regions, with the most substantial decrease evident at T4-P4 (t= -3.65, *p*=0.002) and Fp1-T6 (t=-3.32, *p*=0.006). A similar trend was seen in the alpha band, where the 2-mA group typically had lower PLV values across most regions, with the most pronounced decreases found at T5-Cz (t= -3.62, *p*=0.004) and Pz-C4 (t=-3.50, *p*=0.018). This pattern persisted in the beta band, where the largest decrease in functional connectivity in the 2-mA group compared to the 1-mA group was noted at Fp2-T3 (t= -4.54, *p<*0.001) (Fig. S2).

References

Alipoor A, Nori N (2006) A study on reliability and validity of the Depression-Happiness Scale in justice’s staff of Isfahan city. Journal of Fundamentals of Mental Health 8:85-96.

Beck AT, Steer RA, Brown GK (1996) Beck depression inventory-II. San Antonio 78:490-498.

Beck AT, Epstein N, Brown G, Steer RA (1988) An inventory for measuring clinical anxiety: psychometric properties. Journal of consulting and clinical psychology 56:893.

Criaud M, Boulinguez P (2013) Have we been asking the right questions when assessing response inhibition in go/no-go tasks with fMRI? A meta-analysis and critical review. Neuroscience & Biobehavioral Reviews 37:11-23.

Delorme A, Makeig S (2004) EEGLAB: an open source toolbox for analysis of single-trial EEG dynamics including independent component analysis. J Neurosci Methods 134:9-21.

Eriksen BA, Eriksen CW (1974) Effects of noise letters upon the identification of a target letter in a nonsearch task. Percept Psychophys 16:143-149.

Ghassemzadeh H, Mojtabai R, Karamghadiri N, Ebrahimkhani N (2005) Psychometric properties of a Persian-language version of the Beck Depression Inventory - Second edition: BDI-II-PERSIAN. Depression and Anxiety 21:185-192.

Kane MJ, Conway AR, Miura TK, Colflesh GJ (2007) Working memory, attention control, and the N-back task: a question of construct validity. J Exp Psychol Learn Mem Cogn 33:615.

Lachaux JP, Rodriguez E, Martinerie J, Varela FJ (1999) Measuring phase synchrony in brain signals. Hum Brain Mapp 8:194-208.

Leyfer OT, Ruberg JL, Woodruff-Borden J (2006) Examination of the utility of the Beck Anxiety Inventory and its factors as a screener for anxiety disorders. Journal of Anxiety Disorders 20:444-458.

MacLeod C, Mathews A, Tata P (1986) Attentional bias in emotional disorders. Journal of Abnormal Psychology 95:15-20.

Maust D, Cristancho M, Gray L, Rushing S, Tjoa C, Thase ME (2012) Chapter 13 - Psychiatric rating scales. In: Handbook of Clinical Neurology (Aminoff MJ, Boller F, Swaab DF, eds), pp 227-237: Elsevier.

Moritz S, Von MÜHlenen A, Randjbar S, Fricke S, Jelinek L (2009) Evidence for an attentional bias for washing- and checking-relevant stimuli in obsessive–compulsive disorder. Journal of the International Neuropsychological Society 15:365-371.

Nejat S, Montazeri A, Holakouie Naieni K, Mohammad K, Majdzadeh SR (2006) The World Health Organization quality of Life (WHOQOL-BREF) questionnaire: Translation and validation study of the Iranian version. Journal of School of Public Health and Institute of Public Health Research 4:1-12.

Niso G, Tadel F, Bock E, Cousineau M, Santos A, Baillet S (2019) Brainstorm pipeline analysis of resting-state data from the open MEG archive. Frontiers in neuroscience 13:284.

Owen AM, McMillan KM, Laird AR, Bullmore E (2005) N-back working memory paradigm: A meta-analysis of normative functional neuroimaging studies. Hum Brain Mapp 25:46-59.

Reuman L, Jacoby RJ, Blakey SM, Riemann BC, Leonard RC, Abramowitz JS (2017) Predictors of illness anxiety symptoms in patients with obsessive compulsive disorder. Psychiatry Research 256:417-422.

Ridderinkhof KR, van der Molen MW, Band GP, Bashore TR (1997) Sources of interference from irrelevant information: A developmental study. J Exp Child Psychol 65:315-341.

Rostami R, Kazemi R, Nitsche MA, Gholipour F, Salehinejad MA (2017) Clinical and demographic predictors of response to rTMS treatment in unipolar and bipolar depressive disorders. Clinical Neurophysiology 128:1961-1970.

Schneider W, Eschman A, Zuccolotto A (2002) E-Prime: User's guide: Psychology Software Incorporated.

Simon JR, Berbaum K (1990) Effect of conflicting cues on information processing: the ‘Stroop effect’vs. the ‘Simon effect’. Acta Psychol (Amst) 73:159-170.

Skevington SM, Lotfy M, O'Connell KA (2004) The World Health Organization's WHOQOL-BREF quality of life assessment: Psychometric properties and results of the international field trial. A Report from the WHOQOL Group. Qual Life Res 13:299-310.

Tadel F, Baillet S, Mosher JC, Pantazis D, Leahy RM (2011) Brainstorm: a user-friendly application for MEG/EEG analysis. Comput Intell Neurosci 2011:1-13.

van den Heuvel OA, Veltman DJ, Groenewegen HJ, Witter MP, Merkelbach J, Cath DC, van Balkom AJLM, van Oppen P, van Dyck R (2005) Disorder-Specific Neuroanatomical Correlates of Attentional Bias in Obsessive-compulsive Disorder, Panic Disorder, and Hypochondriasis. Archives of General Psychiatry 62:922-933.

Velloso P, Piccinato C, Ferrão Y, Aliende Perin E, Cesar R, Fontenelle L, Hounie AG, do Rosário MC (2016) The suicidality continuum in a large sample of obsessive–compulsive disorder (OCD) patients. Eur Psychiatry 38:1-7.

Welch P (1967) The use of fast Fourier transform for the estimation of power spectra: A method based on time averaging over short, modified periodograms. IEEE Transactions on Audio and Electroacoustics 15:70-73.

**Figure Legends**

Figure S1: Topographical plots of the relative power changes (post power – pre power) in all frequency bands after each intervention.

Figure S2: Post-intervention functional connectivity across groups

**Tables**

**Table S1**: Means and SDs of reported tDCS side effects.

|  | **sensation** | **Group** | | |  |  |
| --- | --- | --- | --- | --- | --- | --- |
|  |  | **1 mA tDCS** | **2 mA tDCS** | **sham tDCS** |  |  |
|  |  | **M (SD)** | **M (SD)** | **M (SD)** | ***F*** | ***p*-value** |
| **Reported side effect** | Itching |  |  |  |  |  |
|  |  | 0.500 (0.522) | 0.384 (0.506) | 0.333 (0.497) | 0.34 | 0.714 |
|  |  |  |  |  |  |  |
|  | Burning | 1.00 (0.738) | 1.07 (0.640) | 0.833 (0.644) | 0.44 | 0.644 |
|  |  |  |  |  |  |  |
|  | Pain | 0.333 (0.492) | 0.230 (0.438) | 0.250 (0.450) | 0.17 | 0.843 |
|  |  |  |  |  |  |  |
|  | Skin redness | 0.333 (0.492) | 0.461 (0.518) | 0.166 (0.474) | 1.22 | 0.307 |
|  |  |  |  |  |  |  |
|  | Fatigue | 0.500 (0.674) | 0.461 (0.660) | 0.333 (0.602) | 0.24 | 0.786 |
|  |  |  |  |  |  |  |
|  | Trouble concentrating | 0.083 (0.288) | 0.307 (0.480) | 0.083 (0.373) | 1.56 | 0.223 |

Values are presented as means ± standard deviation (SD). Note: each value represents the average of side effects reported during all 10 tDCS sessions. tDCS = transcranial Direct Current Stimulation; M = Mean; SD = Standard Deviation; (*p* ≤ 0.05)

**Table S2**: Means and SDs of clinical outcome measures, and neuropsychological test battery performance before, immediately after, and at 1 month follow-up of the interventions.

| **Measure** | **Outcome variable** | **Time** | **Group M (SD)** | | |  |
| --- | --- | --- | --- | --- | --- | --- |
|  |  |  | **1-mA tDCS** | **2-mA tDCS** | **sham tDCS** | ***p*-value** |
|  |  |  | **M (SD)** | **M (SD)** | **M (SD)** |  |
| **Y-BOCS** | score | Pre-intervention | 25.16 (6.58) | 26.00 (6.84) | 25.25 (7.16) | 0.067 |
|  |  | Post-intervention | 18.58 (7.06) | 18.69 (7.93) | 25.16 (6.36) |  |
|  |  | Follow-up | 17.08 (6.48) | 17.23 (10.15) | 23.83 (4.98) |  |
|  |  |  |  |  |  |  |
| **BAI** | score | Pre-intervention | 24.25 (13.34) | 27.53 (14.89) | 24.41 (13.74) | 0.838 |
|  |  | Post-intervention | 15.41 (8.80) | 15.84 (12.24) | 25.41 (8.03) |  |
|  |  | Follow-up | 18.33 (12.22) | 14.15 (12.34) | 24.08 (8.02) |  |
|  |  |  |  |  |  |  |
| **BDI-II**  **(depression)** | score | Pre-intervention | 29.50 (9.977) | 30.76 (9.90) | 28.33 (7.47) | 0.372 |
|  |  | Post-intervention | 15.08 (7.25) | 18.23 (11.80) | 27.16 (7.40) |  |
|  |  | Follow-up | 15.50 (8.71) | 16.07 (11.87) | 26.91 (7.90) |  |
|  |  |  |  |  |  |  |
| **WHOQUL**  **(quality of life)** | score | Pre-intervention | 68.08 (14.77) | 66.76 (19.38) | 69.33 (15.70) | 0.274 |
|  |  | Post-intervention | 71.75 (9.78) | 79.92 (23.29) | 68.25 (13.22) |  |
|  |  | Follow-up | 68.66 (11.43) | 79.38 (18.200) | 72.16 (11.67) |  |
|  |  |  |  |  |  |  |
| **Flanker test**  **(response inhibition)** | Congruent** | Pre-intervention | 0.754 (0.211) | 0.783 (0.197) | 0.769 (0.322) | 0.960 |
|  |  | Post-intervention | 0.520 (0.103) | 0.688 (0.181) | 0.786 (0.205) |  |
|  |  | Follow-up | 0.733 (0.308) | 0.740 (0.177) | 0.764 (0.293) |  |
|  |  |  |  |  |  |  |
|  | Incongruent ** | Pre-intervention | 0.861 (0.256) | 0.869 (0.255) | 0.878 0.387) | 0.991 |
|  |  | Post-intervention | 0.578 (0.149) | 0.722 (0.154) | 0.808 (0.294) |  |
|  |  | Follow-up | 0.800 (0.258) | 0.760 (0.149) | 0.794 (0.316) |  |
|  |  |  |  |  |  |  |
|  | conflict score | Pre-intervention | 0.105 (0.238) | 0.087 (0.292) | 0.108 (0.242) | 0.997 |
|  |  | Post-intervention | 0.057 (0.215) | 0.034 (0.220) | 0.021 (0.238) |  |
|  |  | Follow-up | 0.067 (0.162) | 0.020 (0.176) | 0.029 (0.194) |  |
|  |  |  |  |  |  |  |
| **Go/No-Go**  **(response inhibition)** | accuracy Go | Pre-intervention | 86.97 (14.34) | 88.98 (10.99) | 89.11 (8.72) | 0.879 |
|  |  | Post-intervention | 92.70 (11.78) | 95.93 (5.29) | 91.13 (5.69) |  |
|  |  | Follow-up | 89.05 (12.46) | 92.15 (11.43) | 91.80 (6.53) |  |
|  |  |  |  |  |  |  |
|  | accuracy NoGo | Pre-intervention | 79.09 (15.06) | 77.78 (16.95) | 83.48 (10.70) | 0.600 |
|  |  | Post-intervention | 85.12 (11.14) | 83.13 (13.29) | 85.23 (8.05) |  |
|  |  | Follow-up | 90.86 (5.65) | 89.66 (8.50) | 83.84 (5.79) |  |
|  |  |  |  |  |  |  |
|  | accuracy NoGo RT* | Pre-intervention | 1.151 (0.196) | 1.157 (0.196) | 1.199 (0.245) | 0.833 |
|  |  | Post-intervention | 1.076 (0.122) | 1.00 (0.145) | 1.193 (0.188) |  |
|  |  | Follow-up | 1.101 (0.161) | 1.013(0.125) | 1.129 (0.143) |  |
|  |  |  |  |  |  |  |
| **Nback**  **(working memory)** | RT** | Pre-intervention | 220.26 (74.35) | 203.91 (50.48) | 197.42 (47.80) | 0.619 |
|  |  | Post-intervention | 178.38 (54.86) | 123.31 (31.06) | 182.81 (37.39) |  |
|  |  | Follow-up | 155.08 (53.15) | 117.68 (36.47) | 183.45 (32.11) |  |
|  |  |  |  |  |  |  |
|  | accuracy | Pre-intervention | 87.41 (15.96) | 84.07 (16.30) | 85.16 (19.00) | 0.885 |
|  |  | Post-intervention | 90.25 (12.87) | 91.00 (8.59) | 86.00 (17.13) |  |
|  |  | Follow-up | 89.00 (13.96) | 90.00 (10.14) | 84.91 (17.69) |  |
|  |  |  |  |  |  |  |
| **Visual probe task (attentional bias)** | OCD stimuli-RT* | Pre-intervention | 1.05 (0.131) | 1.25 (0.313) | 1.06 (0.224) | 0.075 |
|  |  | Post-intervention | 0.994 (0.113) | 1.06 (0.216) | 1.08 (0.208) |  |
|  |  | Follow-up | 1.01 (0.142) | 1.09 (0.249) | 1.08 (0.207) |  |
|  |  |  |  |  |  |  |
|  | neutral stimuli- RT* | Pre-intervention | 1.03 (0.150) | 1.18 (0.265) | 1.07 (0.207) | 0.210 |
|  |  | Post-intervention | 0.938 (0.091) | 0.972 (0.168) | 1.10 (0.201) |  |
|  |  | Follow-up | 0.976 (0.129) | 0.976 (0.111) | 1.13 (0.220) |  |
|  |  |  |  |  |  |  |

*Note*: tDCS = transcranial Direct Current Stimulation; M = Mean; SD = Standard Deviation; Y-BOC = Yale-Brown Obsessive-Compulsive Scale; BAI = Beck Anxiety Inventory; BDI-II = Beck Depression Inventory-II; WHOQUL = WHO Quality of Life; RT = Reaction time; Values marked by (*) and (**) are given in seconds and milliseconds respectively. *p* values refer to between-group baseline (i.e., pre-intervention) comparisons by ANOVA tests.
