# Supplementary figures and images for: Targeting the prefrontal-supplementary motor network in obsessive-compulsive disorder with intensified electrical stimulation in two dosages: a randomized, controlled trial
Source: Transl Psychiatry. 2024 Feb 5;14:78. doi: 10.1038/s41398-024-02736-y (PMC10844238; doi:10.1038/s41398-024-02736-y)

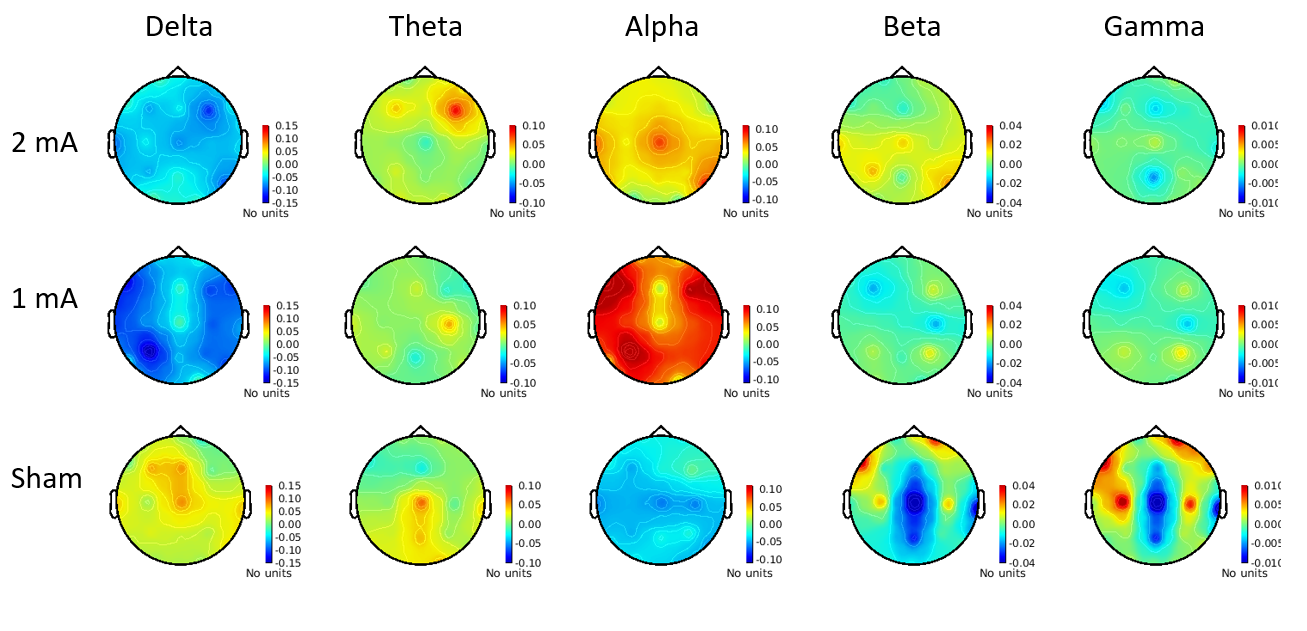

Supplement: Supplementary file 2 — Figure S1 [file 41398_2024_2736_MOESM2_ESM.bmp]
