# Supplementary material for: Targeting the prefrontal-supplementary motor network in obsessive-compulsive disorder with intensified electrical stimulation in two dosages: a randomized, controlled trial
Source: Transl Psychiatry. 2024 Feb 5;14:78. doi: 10.1038/s41398-024-02736-y (PMC10844238; doi:10.1038/s41398-024-02736-y)

1-mA group Vs. Sham

non-parametric permutation independent t-test on ***Post-Intervention*** *condition results*

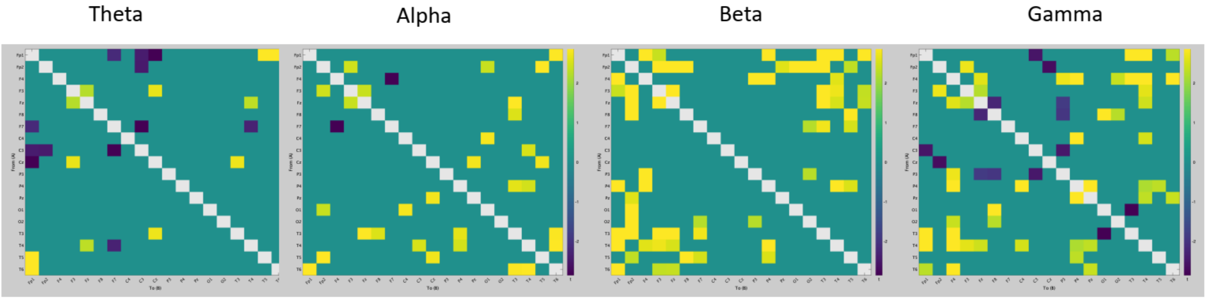

2-mA group Vs. Sham

non-parametric permutation independent t-test on ***Post-Intervention*** *condition results*

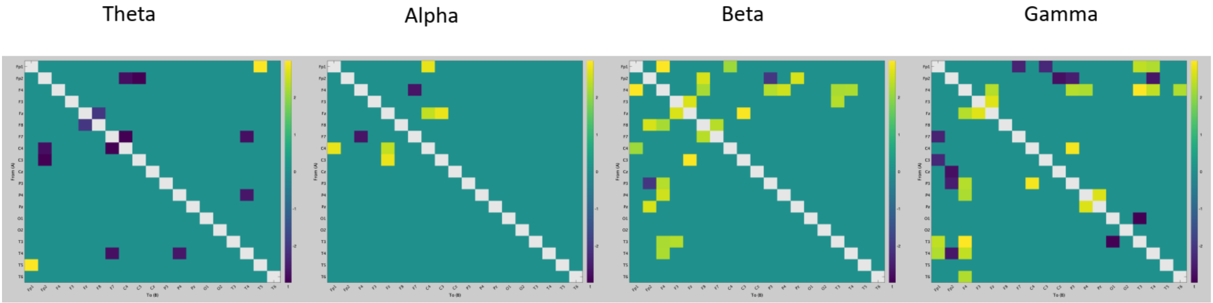

2-mA group Vs. 1-mA group

non-parametric permutation independent t-test on ***Post-Intervention*** *condition results*

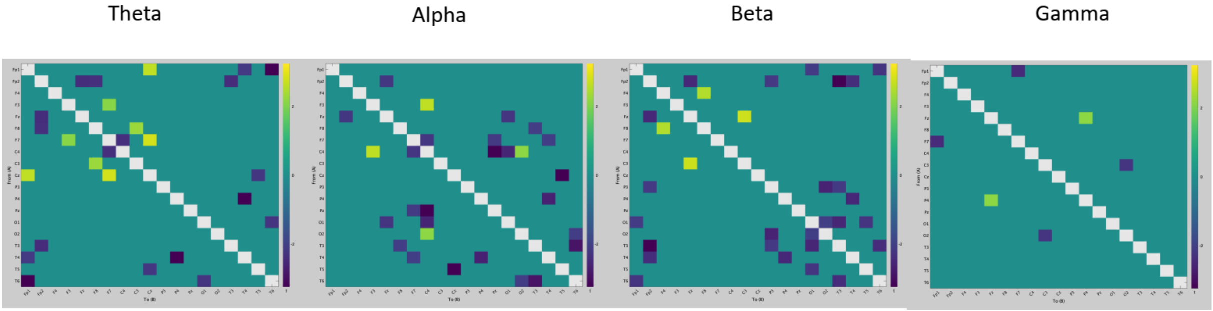

Supplement: Supplementary file 3 — Figure S2 [file 41398_2024_2736_MOESM3_ESM.pdf]
